# Genome-wide identification of and functional insights into the late embryogenesis abundant (*LEA*) gene family in bread wheat (*Triticum aestivum*)

**DOI:** 10.1038/s41598-019-49759-w

**Published:** 2019-09-16

**Authors:** Hao Liu, Mingyan Xing, Wenbo Yang, Xiaoqian Mu, Xin Wang, Feng Lu, Yao Wang, Linsheng Zhang

**Affiliations:** 1 College of Life Science/State Key Laboratory of Crop Stress Biology for Arid Areas, Northwest A&F University, Yangling, China; 2 Cereal Crops Research Institute, Henan Academy of Agricultural Sciences, Zhengzhou, China; 3 College of Life Science, Jinan University, Guangzhou, China

**Keywords:** Agricultural genetics, Transcription

## Abstract

Late embryogenesis abundant (LEA) proteins are involved in the responses and adaptation of plants to various abiotic stresses, including dehydration, salinity, high temperature, and cold. Here, we report the first comprehensive survey of the LEA gene family in “Chinese Spring” wheat (*Triticum aestivum*). A total of 179 *TaLEA* genes were identified in *T*. *aestivum* and classified into eight groups. All *TaLEA* genes harbored the LEA conserved motif and had few introns. *TaLEA* genes belonging to the same group exhibited similar gene structures and chromosomal locations. Our results revealed that most *TaLEA* genes contained abscisic acid (ABA)-responsive elements (ABREs) and various *cis*-acting elements associated with the stress response in the promoter region and were induced under ABA and abiotic stress treatments. In addition, 8 genes representing each group were introduced into *E*. *coli* and yeast to investigate the protective function of TaLEAs under heat and salt stress. TaLEAs enhanced the tolerance of *E*. *coli* and yeast to salt and heat, indicating that these proteins have protective functions in host cells under stress conditions. These results increase our understanding of LEA genes and provide robust candidate genes for future functional investigations aimed at improving the stress tolerance of wheat.

## Introduction

Abiotic stress, such as drought, high temperature, cold, and salinity, is a primary factor of reduced crop productivity due to serious disruptions in plant growth and development^1^. Therefore, plants have evolved complex regulatory mechanisms in their defense responses to adverse conditions. All mechanisms are based on proteins that directly function in abiotic tolerance and that regulate various signaling pathways to indirectly improve abiotic tolerance. The late embryogenesis abundant (LEA) protein gene family is an important group of functional proteins to reduce cell damage and protect cells under abiotic stress conditions^2,3^.

LEA genes were first observed in late-stage mature cotton seeds^4^. Since then, they have been detected in seedlings, leaves, stems, roots and other organs of many other plants mostly under abiotic stress conditions that result in cellular dehydration^5,6^. LEA genes have also been identified in bacteria and invertebrates^7,8^. LEA proteins accumulate during late embryonic development and can be induced by various abiotic stresses^9^. Compared with other proteins involved in abiotic stress tolerance, LEA proteins have no significant enzymatic activity; however, LEA proteins, as intrinsically disordered proteins (IDPs), are highly hydrophilic and intrinsically unstructured in the hydrated state but partially fold into mainly *α*-helical structures under dehydration conditions^5,7^. This feature allows them to function as chaperones via preventing protein aggregation during abiotic stress^10,11^. In addition, LEA proteins contribute to the stabilization of membranes, binding of calcium and metal ions, interactions between DNA and RNA, and the protection of functional proteins against aggregation^12–15^.

Based on conserved motifs, amino acid sequences and phylogenetic relationships, LEA proteins are essentially classified into eight groups: LEA_1, LEA_2, LEA_3, LEA_4, LEA_5, LEA_6, dehydrin (DHN) and seed maturation protein (SMP)^16–18^. Genome-wide characterizations of LEA family proteins have been performed in several plant species, such as *Arabidopsis thaliana*^18^, *Brassica napus*^19^, *Citrus sinensis*^20^, *Dendrobium officinale*^21^, *Hordeum vulgare*^22^, legumes^23^, *Malus domestica*^24^, *Oryza sativa*^25^, *Pinus tabuliformis*^26^, *Populus trichocarpa*^27^, *Prunus mume*^28^, *Solanum tuberosum*^29^, and *Zea mays*^30^. However, few LEA genes have been identified in wheat.

Recently, the chromosome-based draft genome of the bread wheat “Chinese Spring” was published^31^, making the identification and analysis of the LEA gene family in wheat possible. Therefore, in the present study, we performed a genome-wide analysis of LEA genes in wheat to characterize their sequences, evolutionary relationships, putative functions and expression patterns in response to different abiotic stresses.

## Results

### Sequences and characteristics of the *TaLEA* genes

**Figure 1:**
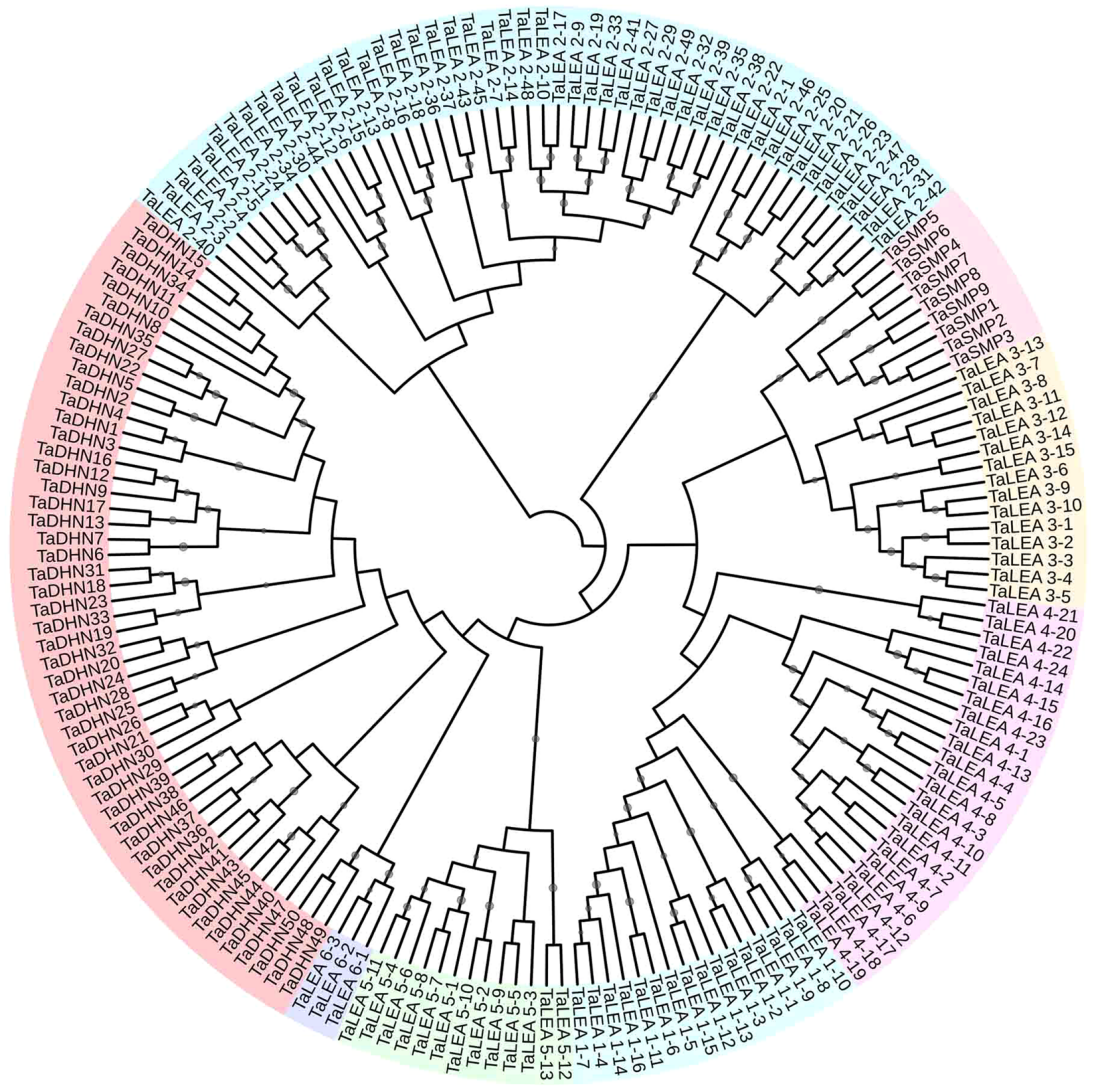
Phylogenetic tree of TaLEA proteins in wheat. The Maximum Likelihood (ML) tree was generated using MEGA7 with 1000 bootstrap replicates. LEA gene families are distinguished by different colors.

A total of 179 LEA proteins were identified from wheat (Table S5) based on a Pfam ID search of wheat genome databases and homologous sequence alignment with LEA genes from *A*. *thaliana* and *T*. *aestivum*. The *TaLEA* genes were classified into eight subfamilies based on their conserved domains (Fig. 1). The LEA_2 and DHN families were the largest, with 49 and 50 members, respectively. The LEA_1, LEA_3, LEA_4, LEA_5, and SMP families included 16, 15, 24, 13, and 9 members, respectively. The LEA_6 family included only 3 members.

The *TaLEA* genes encode polypeptides of 89–1062 amino acids in length, with predicted molecular weights ranging from 9.1 to 108.7 kDa. An analysis of their physicochemical properties (Table S5) revealed that 112 LEA proteins (63%) have relatively high isoelectric points (pI > 7). The remaining 67 proteins have pI < 7, including all LEA_5 and LEA_6 proteins and some members of other families. The calculated grand average of hydropathy index (GRAVY) values suggested that 151 proteins (84%) are quite hydrophilic and that 28 members (16%) are hydrophobic, with the latter group of proteins all belonging to the LEA_2 family. Subcellular localization prediction indicated that LEA_1, LEA_5, LEA_6, and SMP proteins are located exclusively in the nucleus; most of the LEA_2 and LEA_3 proteins are in the chloroplast; LEA_4 proteins are in the cell wall and nucleus; and DHN proteins are in the cytoplasm and nucleus.

### Structural characterization of the *TaLEA* genes

**Figure 2:**
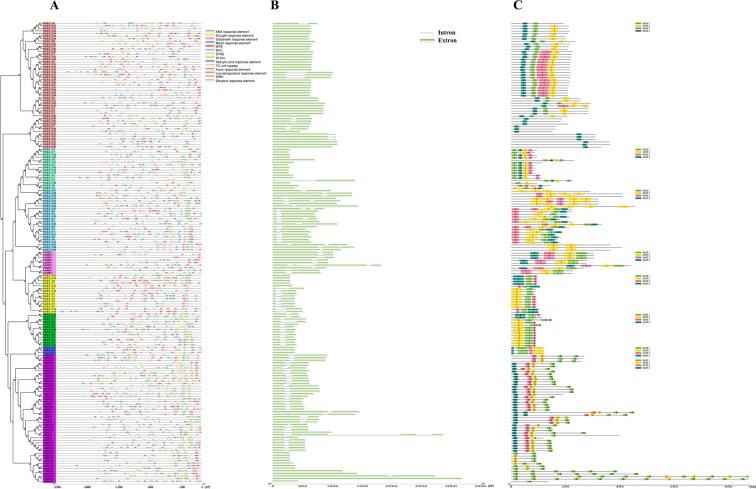
*Cis*-acting elements on promoters (**A**) exon–intron organization (**B**) and conversed motif in the different groups (**C**) of the *TaLEA* genes.

To investigate the structural characteristics of the LEA genes, the exon-intron structures (Fig. 2B) and conserved motifs (Fig. 2C) of 179 *TaLEA* genes were analyzed. The majority of the *TaLEA* genes contain zero or one intron, and only 6 *TaLEA* genes (*TaLEA_4-1*, *TaLEA_4-20*, *TaLEA_4-21*, *TaLEA_4-23*, *TaSMP4*, and *TaDHN5*) have two or three introns (Fig. 2B). Members of each *TaLEA* subfamily have similar exon-intron structures, and TaLEAs with closer evolutionary relationships had more similar numbers and lengths of introns and exons.

Motif analysis indicated that 179 TaLEA proteins lack high similarity (Fig. S2). Nevertheless, members of each LEA subfamily have several conserved group-specific domains (Figs 2C and S3). For example, motif 4 of LEA_2, which is present in all TaLEA_2 proteins, encodes a conserved LEA domain. The K-segment (motif 1 and motif 4 of the DHN group), an important conserved motif in the DHN family, exists in all DHNs. Interestingly, DHN members except YnKn- and KnS-type DHNs induced only YnSKn-, SKn- and Kn-type DHNs in wheat. Most of the closely related genes in the evolutionary relationships of each family exhibit similar motif compositions, suggesting functional similarities in the LEA family. These results indicate that the composition of the structural motifs varies among different LEA families but is similar within families and that the motifs encoding the LEA domains are conserved.

### Chromosomal locations of the *TaLEA* genes

**Figure 3:**
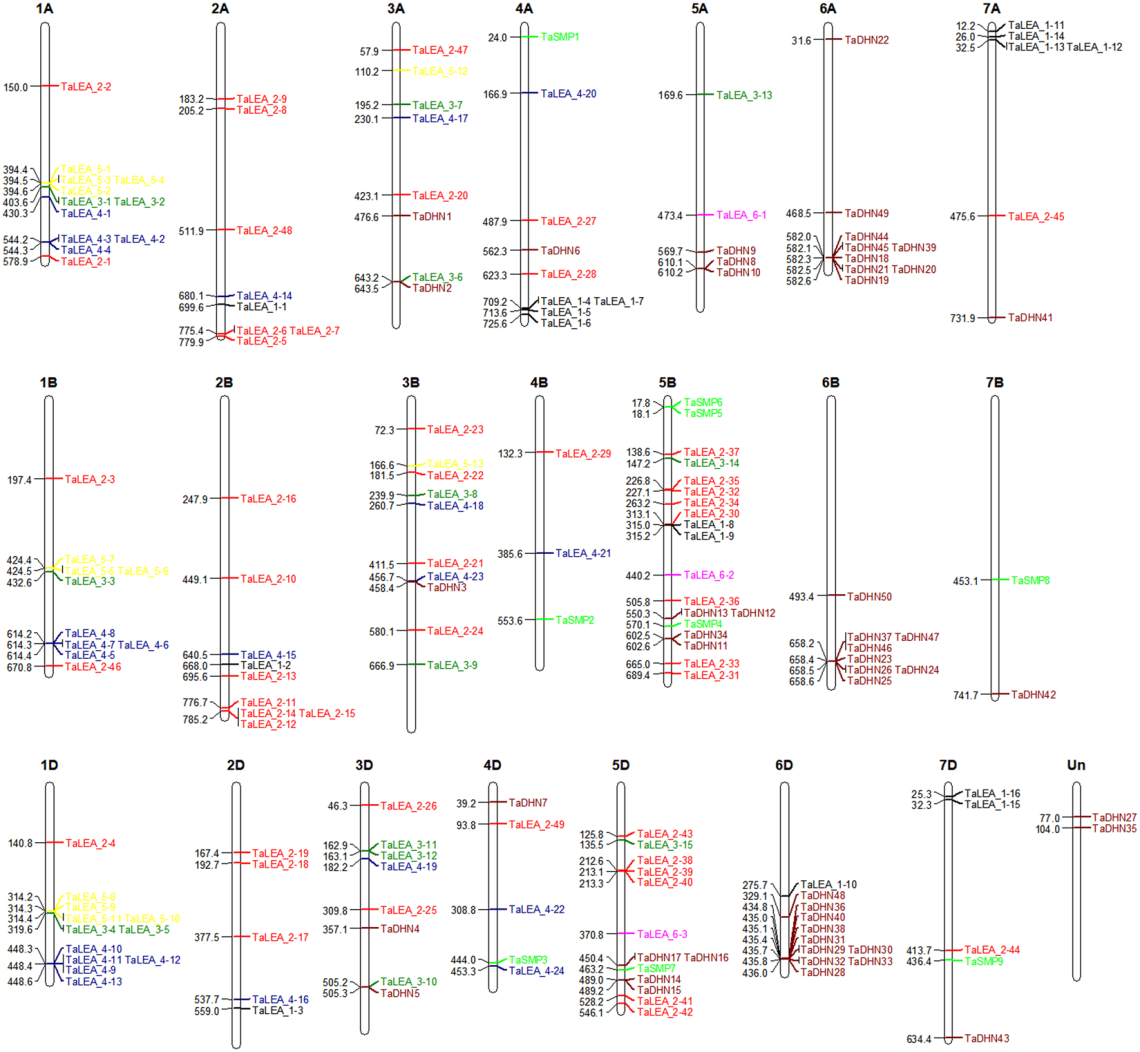
Chromosome distributions of *TaLEA* genes in wheat. LEA gene subfamilies are distinguished by different colors.

The chromosomal locations of the identified *TaLEA* genes in wheat were mapped to the corresponding chromosomes by the MapChart tool. Of the 179 *TaLEA* genes, 177 could be mapped to the assembled chromosome, whereas two genes (*TaDHN27* and *TaDHN35*) were distributed across an unassembled scaffold (Fig. 3 and Table S5). *TaLEA*s were extensively and unevenly distributed on different chromosomes. Chromosomes 5B contained the largest number of *TaLEA* genes, with 19 genes. In contrast, only two were annotated on chromosomes 7B. *TaLEA* genes were approximately evenly and similarly distributed in the A (57), B (61), and D (59) subgenomes. For example, the 1A, 1B and 1D chromosomes had similar numbers of LEA_2, LEA_3, LEA_4, and LEA_5 genes, and the evolutionary relationships in each subfamily were close (Fig. 1). In addition, different LEA subfamilies had different gene distributions on different chromosomes. The LEA_1 genes were distributed on chromosomes 2, 4, 5, 6, and 7. The LEA_2 genes were distributed on all chromosomes except chromosome 6. LEA_3s were mainly distributed on chromosomes 1, 3 and 5. LEA_4s were distributed on chromosomes 1, 2, 3, and 4, and LEA_5s were distributed on chromosomes 1 and 3. SMPs were distributed on chromosomes 4, 5, and 7. DHNs were distributed on chromosomes 3, 4, 5, 6, and 7. Thus, the chromosomal locations of *TaLEA* genes in wheat may be caused by LEA gene duplication patterns.

### Analysis of *cis*-acting elements in the promoter of the *TaLEA* genes

The presence of multiple different *cis*-acting elements in the gene promoters may indicate that these genes perform different functions. To explore the *cis*-acting elements in the *TaLEA* gene promoters, we extracted approximately 2 kb of the upstream genomic sequence of each gene transcription start site (TSS) and then searched the PlantCARE database to identify and count the *cis*-acting elements associated with plant growth and development and stress response (Fig. 2A and Table S6). For *cis*-acting regulatory elements associated with plant hormone responses, abscisic acid (ABA)-responsive elements (ABREs) (92.7%), which respond to ABA and regulate downstream gene expression, were found in most *TaLEA* gene promoters. In addition, *TaLEA_3-8* had the most (11) ABREs in its promoter. CGTCA motifs and TGACG motifs involved in the methyl jasmonate (MeJA) response were also identified, accounting for approximately 67.6%. There were also a few *TaLEA* gene promoters that included auxin response elements (TGA-elements), ethylene response elements (EREs), gibberellin response elements (GAREs), and salicylic acid response elements (TCA-elements). At the same time, we also identified a large number of *cis-*acting elements related to plant stress response, MYB (91.6%), MYC (64.2%), W box (27.4%) and TC-rich repeats (10.6%), which can participate in a variety of plant responses to abiotic stress. DRE, LTRE and STRE are capable of responding to drought, low-temperature and heat stress, respectively. WRE is associated with plant damage and infection by pathogens. These results indicate that the TaLEA genes may participate in the growth and development of plants through the ABA pathway and respond to various stresses.

### Determining the endogenous wheat ABA content and expression patterns of *TaLEA* genes under ABA and abiotic stress treatments

**Figure 4:**
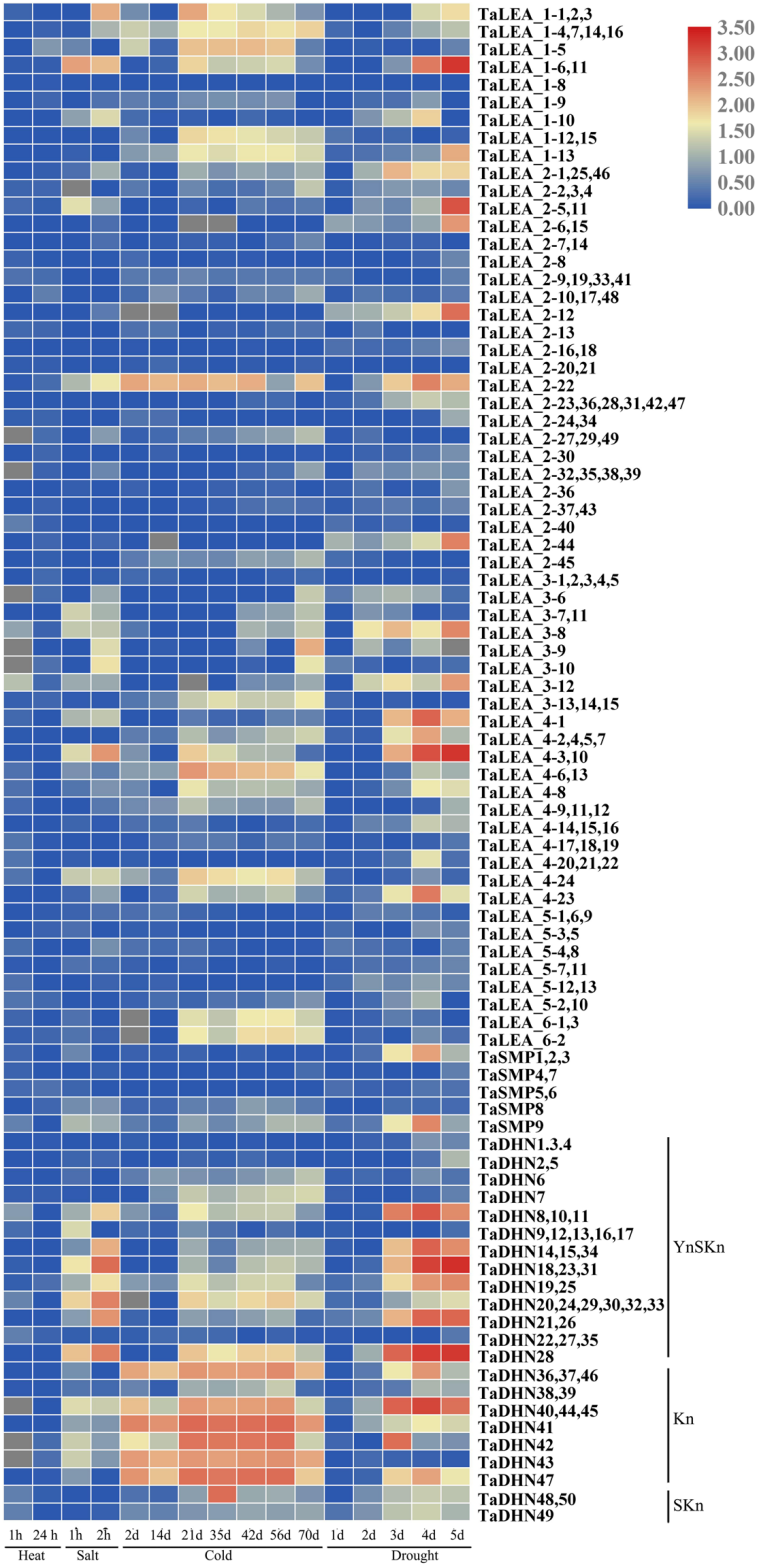
Expression profiles of the *TaLEA* genes in wheat. Dynamic expression profiles of *TaLEA* genes high temperature, salt, cold and drought treatments using publicly available microarray data.

To investigate the expression patterns of the *LEA* genes in wheat under abiotic stress, a microarray analysis of *TaLEA* genes was carried out (Fig. 4 and Table S7). We found that all *TaLEA* genes were expressed in at least one of the stress treatments tested, and these genes displayed various expression patterns. In addition, 139 *TaLEA* genes were responsive to all stresses. Under cold stress, the expression of 157 *TaLEA* genes was induced. Among these, 46 genes were upregulated by cold stress, with upregulation ranging from 2.01–5.96-fold. *TaDHN41* had the highest expression level after 56 days under cold stress. A total of 177 *TaLEA* genes were induced by drought. Among these, 50 *TaLEAs* were upregulated by drought stress, with a 2.02–8.87-fold upregulation. The expression of *TaDHN18*, *TaDHN23*, and *TaDHN31* was highest after 5 days under drought stress. Twenty-five *TaLEA* genes were induced by high salinity, with a 2.09–5.74-fold upregulation. Under high-temperature stress, there was no obvious change in the expression of most *TaLEA* genes.

**Figure 5:**
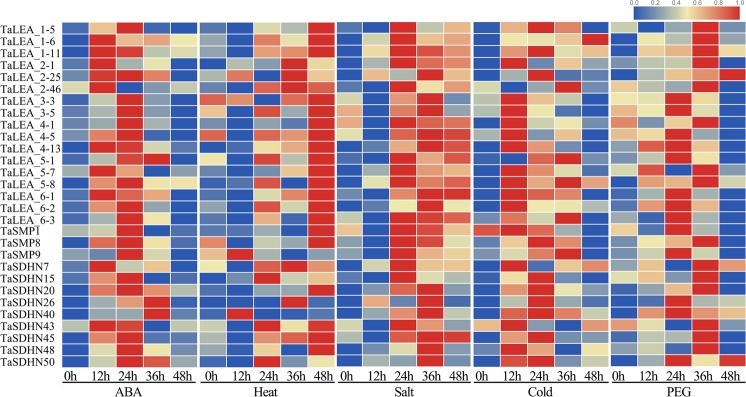
Expression of *TaLEA* genes in response to ABA, heat, NaCl, cold, and PEG treatments determined by real-time PCR. The expression level of wheat *actin* was used as the internal control to standardize the RNA samples for each reaction. The values are the mean ± SE from three samples.

To confirm the results of the microarray data, we selected 29 *TaLEA* genes belonging to LEA_1, LEA_2, LEA_3, LEA_4, LEA_5, LEA_6, SMP and three DHN subfamilies to investigate the expression patterns of *TaLEA* genes in wheat seedlings subjected to ABA and abiotic stress treatments by real-time PCR (Fig. 5). The expression of all *TaLEA* genes was upregulated with ABA, cold, PEG, salt and heat treatments. Interestingly, *cis*-acting elements that responded to ABA were not identified in the promoter of *TaSMP1* (Fig. 2A); however, *TaSMP1* was still induced by ABA, probably due to the lack of the 673–1017 bp upstream genomic sequence of the *TaSMP1* TSS. These results suggested that they may play vital roles under these stress conditions.

**Figure 6:**
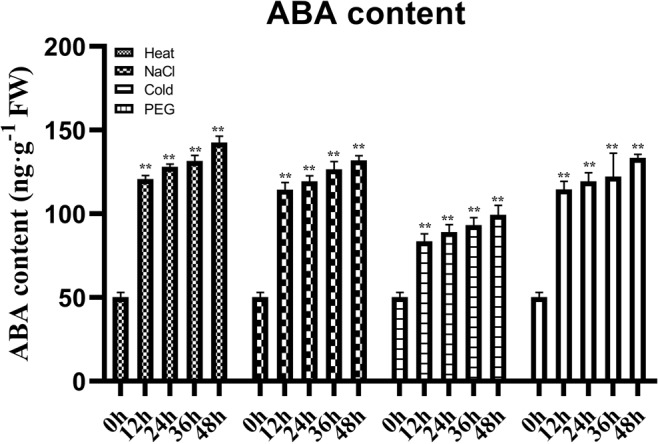
Endogenous ABA content in wheat leaves under heat, NaCl, cold, and PEG stresses as determined by ELISA. The values are the mean ± SE from three samples and significant differences were indicated as ^(*)^p < 0.05, and ^(**)^p < 0.01.

We also determined the endogenous ABA content in wheat leaves under different stress treatments (Fig. 6). The ABA content in wheat leaves increased gradually under PEG, high-salt, low-temperature and high-temperature treatments from 0 h to 48 h. In contrast, the expression most *TaLEA* genes were downregulated at 48 h under cold, PEG and salt stress. Thus, we speculated that these genes were regulated via two pathways: one that requires ABA directly and another that is induced by osmotic stress independently of ABA synthesis.

### Enhancement of the tolerance of recombinant *E*. *coli* and yeast cells to salt and heat-

To determine the function of TaLEA proteins in stress conditions, we selected one gene from each LEA group, transformed the gene into *E*. *coli* and yeast to construct the recombinants pET28a-*TaLEA* or pPI3.5k*-TaLEA*, and carried out salt and heat treatments.

For the salt treatment, *E*. *coli* strains carrying 5 *TaLEA* genes had mean viability ratios 2-7-fold higher than those of the control strain under heat stress (Fig. 5A). Among these genes, TaLEA_3-3 (~7.01-fold), TaLEA_4-1 (~6.45-fold), TaLEA_6-2 (~1.94-fold) and TaDHN43 (~4.24-fold) had significantly higher survival ratios than other genes under different concentrations. In addition, strains carrying the *TaLEA* genes showed almost no growth at 500 mM and 600 mM NaCl treatments. Similarly, yeast transformed with pPI3.5k-*TaLEA_3-3*, *TaLEA_4-1*, *TaLEA_6-2*, *TaSMP8* and *TaDHN43* showed better growth than the control (pPI3.5k).

For the heat treatment, our results demonstrated that *E*. *coli* strains carrying six (*TaLEA_1-5*, *TaLEA_3-3*, *TaLEA_4-1*, *TaLEA_5-1*, *TaSMP8*, and *TaDHN43*) of the eight *TaLEA* genes had mean viability ratios 2-3-fold higher than those of the control strain under salt stress (Fig. 5B). Among these genes, *TaDHN43* (~2.74-fold) showed the highest viability ratio after induction at 50 °C for 30 min. After they were induced at 50 °C for 60 min and 120 min, *TaLEA_1-5* (~1.98-fold) and *TaLEA_3*-*3* (~2.76-fold) had the highest viability ratios, respectively. In addition, the viability ratio of TaLEA_1-5, TaLEA_3-3, TaLEA_4-1, TaLEA_5-1, and TaDHN43 was higher than that of the control strain after induction at 50 °C for 180 min. There were no differences in the mean viability ratios between other *TaLEA* genes and the control strain. Consistent with response of the *E*. *coli* recombinants under heat stress, the yeast transformed with pPI3.5k-*TaLEA_1-5*, *TaLEA_3*-*3*, *TaLEA_4-1*, *TaLEA_5-1*, *TaSMP8*, and *TaDHN43* showed better growth than the control, and the transformed yeast reached the stationary phase faster than the control.

## Discussion

### Molecular characteristics and phylogenetics of the wheat LEA gene family

*LEA* genes play crucial roles in embryonic development and in response to abiotic stress^5^. The *LEA* gene family has been reported in many crops; in contrast, genome-wide identification and annotation of *LEA* genes have not been reported in *T*. *aestivum*. In this study, 179 *TaLEA* genes were identified in the *T*. *aestivum* genome (Table S1), which is the most abundant *LEA* gene family identified in plants so far. Based on phylogenetic analyses, these 179 *TaLEA* genes belong to eight groups of the LEA gene family.

Motif analysis of the TaLEA proteins showed that members of each LEA group contained specific conserved motifs (Fig. 2) that have been previously identified in several plant species, such as *A*. *thaliana*^18^, *D*. *officinale*^21^ and *P*. *trichocarpa*^27^. The conserved motifs observed within each LEA group determine the probable origin within the groups and indicate that the TaLEA proteins have group-specific functions.

In recent years, some research has reported that stress-response genes generally contain relatively few introns^27^. Notably, 173 of the 179 (97%) *TaLEA* genes have less than one intron. Low intron numbers have also been observed in other stress-response gene families, such as the glutathione transferase family^27^. Introns can have a deleterious effect on gene expression by delaying transcript production. Introns can delay regulatory responses by extending the length of the nascent transcript, resulting in an additional energetic cost due to increased transcript length^32^.

Subcellular localization analyses showed the presence of TaLEA proteins in all subcellular compartments, including the nucleus, cell membranes, cell walls, mitochondria, chloroplasts, Golgi apparatus, and cytoplasm, as also reported for *Arabidopsis*^18^ and tomato^33^. There is a strong implication that LEA proteins from the principal groups are ubiquitous within cells and their respective tissues, suggesting that their function is required in all cellular compartments during stress^34^.

### Expression and function of wheat LEA genes in response to abiotic stress

Analysis of the physicochemical properties revealed that the TaLEA proteins had common characteristics, such as small molecular weights and rich hydrophilic amino acids (Table S5), as reported in *D*. *officinale* and *P*. *tabuliformis*^21,26^. Most LEA proteins are predicted to have no stable secondary structure in solution, i.e., IDPs, but they may acquire α-helical structures upon dehydration^35^. This characteristic of LEA proteins allows them to change their conformation according to the changes in their ambient microenvironment, resulting in multiple functions in abiotic stress^10^.

Many studies have shown that LEA genes play an important role in abiotic stress, such as low-temperature, drought and high-salinity stress^36^. Moreover, LEA gene subgroups have evolved different adaptive effects against abiotic stress, as reported in *D*. *officinale* and *P*. *tabuliformis*^21,26^. According to the results of the microarray analysis, the response of most *TaLEA* genes to salt, cold and drought stress was obvious, and genes were especially significantly expressed in the *TaDHN* group, followed by the *TaLEA_1* and *TaLEA_4* groups (Fig. 4 and Table S7). The expression of most *TaLEA* genes had no obvious difference under heat stress, probably due to a lack of adequate induction time data in public databases. These results indicate that the DHN group is a valuable genetic resource in stress resistance research.

The expression patterns of the *TaLEA* genes in wheat under low-temperature, high-temperature, salt, and osmotic stress were analyzed by real-time PCR (Figs 4 and S4). These *TaLEA* genes were all upregulated in response to various stresses, and *TaLEA* genes with closely related evolutionary relationships had similar expression patterns. In potato, most *StLEA* genes from the LEA_1, LEA_2, LEA_3, LEA_6, ASR, and DHN groups were upregulated after low-temperature, high-temperature, salt and drought treatments^37^. In rice, the expression of genes in the LEA1, LEA2, LEA3 and DHN groups was upregulated under osmotic stress and salt stress^38,39^, while in tomato, the expression of genes in the LEA1, LEA2, LEA4, and DHN groups was upregulated after drought and salt stress^33^. These results indicate that nearly all LEA family genes can be induced by various abiotic stresses.

**Figure 7:**
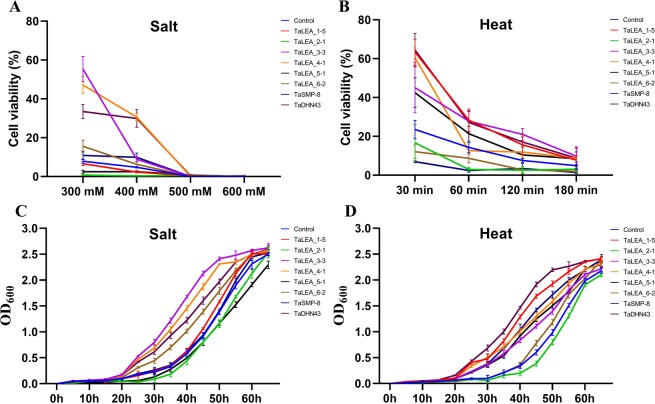
Overexpression enhances tolerance to salt and heat stresses in recombinant *E*. *coli* and yeast cells. Cell Viability ratio of *E*. *coli* transformed with pET28a-*TaLEA* and pET28a (as control group) under salt (**A**) and heat (**B**) treatments. Growth curves of yeast transformed with Ppic3.5K-*TaLEA* and Ppic3.5K (as control group) under salt (**C**) and heat (**D**) treatments. The values are the mean ± SE from three samples.

Previously, functional expression screening of LEA proteins from various plant species was successfully performed in *E*. *coli* and yeast exposed to abiotic stress. Here, we demonstrated that recombinants expressing LEA_3, LEA_4, and DHN proteins grew and survived better under salt and high-temperature stress than *E*. *coli* and yeast not expressing LEA proteins (Fig. 7). In addition, LEA_6 proteins enhanced the tolerance of *E*. *coli* and yeast cells to salt, and LEA_1, LEA_5, SMP proteins enhanced the tolerance of host cells to high temperature. DHNs and their respective truncated derivatives with at least one K-segment obviously ensured *E*. *coli* stabilization under desiccation, high-temperature, and cold treatments^40,41^. Jie Gao *et al*.^40,41^ and Hong Ling *et al*.^21^ investigated most LEA groups except LEA_6 and determined that these LEA groups enhanced *E*. *coli* tolerance to salt and high temperature, whereas some LEA_3 and SMP group members could not improve *E*. *coli* tolerance under salt and high-temperature stress. These results indicate that *LEA* genes have evolved different adaptive functions and play important roles in plants against various abiotic stresses.

## Methods

### Identification of the LEA family genes in the wheat genome

LEA genes were identified in *Triticum aestivum* based on homology with 51 *Arabidopsis* LEA protein sequences and 54 wheat DHN EST contigs via a BLAST search against the wheat genomic database on Phytozome (https://phytozome.jgi.doe.gov/pz/portal.html) and Ensembl (http://plants.ensembl.org/Triticum_aestivum/Info/Index) servers. The open reading frames of *TaLEA* genes were repredicted and corrected using Fgenesh and Fgenesh + software (http://www.softberry.com) in the wheat database. In addition, we also identified LEA proteins in wheat protein sequence data using the BLAST HMM profile (http://pfam.xfam.org) of LEA_1 (PF03760), LEA_2 (PF03168), LEA_3 (PF03242), LEA_4 (PF02987), LEA_5 (PF00477), LEA_6 (PF10714), SMP (PF04927) and DHN (PF00257). All of the identified *TaLEA* candidates were analyzed using the Hidden Markov Model of the Pfam database (http://www.ebi.ac.uk/Tools/hmmer/) and the NCBI Conserved Domain database (https://www.ncbi.nlm.nih.gov/cdd) to confirm the conserved domains of the LEA proteins. Finally, we retrieved 179 *TaLEA* genes classified into 8 LEA protein subfamilies.

### Phylogenetic relationships, gene structures, conserved motifs and chromosomal locations of the *TaLEA* genes

The phylogenetic tree was constructed by the maximum-likelihood method with 1000 bootstrap replicates in MEGA 7.0 software^42^. The exon-intron structures were identified using the Gene Structure Display Server (GSDS) (http://gsds.cbi.pku.edu.cn/) by comparing CDSs and genomic DNA sequences^43^. Multiple EM for Motif Elicitation (MEME) (http://meme.sdsc.edu/meme) was used to identify the conserved motifs encoded by the *TaLEA* family genes^44^. The chromosomal locations of each *TaLEA* gene were mapped to each chromosome according to their positions in the wheat genome. The identified positions were then marked on the chromosomes using the MapChart tool^45^.

### Promoter sequences and microarray-based expression pattern analysis

The promoter sequences, which were approximately 2000 bp upstream of the TSS of the *TaLEA* genes, were acquired from the wheat database, and the *cis*-elements in the promoters were analyzed with the PlantCARE database^46^.

The coding sequences of *TaLEA*s were submitted to the Plex database (http://www.plexdb.org/) to search for corresponding probes. These probes were then used as queries to obtain the expression data of the *TaLEA* genes in GeneVestigator software (https://genevestigator.com/gv/).

### Plant materials and treatments

Wheat seeds of “Chinese Spring” were germinated on moist filter paper at 25/18 °C (day/night) with a photoperiod of 16 h/day. For abiotic stress treatment, seedlings grown in hydroponic culture for two weeks were exposed to 100 μM ABA, high temperature (42 °C), high salinity (800 mM NaCl), cold (4 °C), and 20% PEG 6000 (w/v), as described previously^47^. In each treatment, the leaf tissues were collected every 12 h for 48 h, frozen in liquid nitrogen, and stored at −80 °C.

### RNA isolation and real-time PCR analysis

RNApure Plant Kit (CWBIO) was used to isolate total RNA from each frozen sample, and first-strand cDNA was synthesized from total RNA (1 μg) by using PrimeScript™ II 1st Strand cDNA Synthesis Kit (TaKaRa) according to the manufacturer’s instructions. The sequence was amplified using gene-specific primers (Table S2) with TransTaq-T DNA Polymerase (TransGen), and the *Actin* gene was used as an internal control. The real-time PCR cycling parameters were 94 °C for 30 s, followed by 45 cycles at 94 °C for 5 s and 55 °C for 30 s, with a melting curve analysis from 60 °C to 90 °C at a rate of 0.5 °C/5 s. All reactions were performed in triplicate to ensure the reproducibility of the results.

### Endogenous ABA content assays

The endogenous ABA content of wheat leaf tissues was measured using an enzyme-linked immunosorbent assay (ELISA), as described previously^48^.

### *In vivo* assay of the stress tolerance of transformed *E*. *coli*

We selected 8 *TaLEA* genes representing each group, inserted the genes into the pET28a vector and then transformed the vectors into the *E*. *coli* host strain BL21 (DE3). The primers are shown in Supplementary Table S3. Recombinant proteins were induced with 1 mM isopropyl β-D-1-thiogalactopyranoside (IPTG) for 6 h when recombinant *E*. *coli* BL21 (DE3) density reached an OD_600_ of 0.6 in LB liquid medium containing 50 μg/mL kanamycin at 37 °C. The *E*. *coli* cells were harvested by centrifugation, resuspended in phosphate buffered saline (PBS), and then analyzed by SDS-PAGE (Fig. S1).

Heat and salt tolerance assays were performed as described previously^48^. IPTG-induced *E*. *coli* (pET28a-*TaLEA*) cell cultures were diluted to the same OD_600_ value, transferred to 50 °C, sampled at 60, 120, 180 and 180 min, and plated (20 μL) onto LB plates. For the salt treatment, after IPTG induction, 20 μL of each sample (same OD_600_ value) was spotted onto LB plates containing 300 mM, 400 mM, 500 mM, and 600 mM NaCl. The viability ratio of the transformants under heat and salt conditions was calculated by counting the number of colonies after incubation of the plate overnight at 37 °C. Cell viability ratio = (colony number on stressed plate/colony number on unstressed plate) × 100%. For all experiments (heat and salt), the means of three experiments were determined from three independent transformants. Here, *E*. *coli* with the empty vector (pET28a) was the control group.

### *In vivo* assay of the stress tolerance of transformed yeast

The eight selected *TaLEA* genes were inserted into the Ppic3.5 K vector and then transformed into the *Pichia* yeast strain GS115, as described previously^49–51^. The primers are shown in Supplementary Table S4. Recombinant yeast cells were inoculated into buffered glycerol-complex medium (BMGY; 1% yeast extract, 2% peptone, 1.34% yeast nitrogen base (YNB), 10 mM K_3_PO_4_, 4 × 10^−5^ mM biotin, and 1% glycerin) to induce *TaLEA* gene expression at 30 °C until the stationary phase of growth was reached. Then, the cells were collected by centrifugation and resuspended in 200 mL induction buffered methanol-complex medium (BMMY; 1% yeast extract, 2% peptone, 10 mM K_3_PO_4_, 1.34% YNB, 4 × 10^−5^ mM biotin, and 0.5% methanol) and then incubated at 30 °C for 4 days. Methanol was added every 24 h to a final concentration of 0.5%. Cell cultures of induced yeast (Ppic3.5K-*TaLEA*) were diluted to the same OD_600_ value of 0.8, and 1 mL cells was inoculated into 150 mL of BMGY medium containing 1.2 M NaCl and then induced at 30 °C. For heat stress treatments, 1 mL cells was inoculated into 150 mL of BMGY medium and induced at 40 °C. Growth was monitored with a spectrophotometer by measuring the OD_600_ every 5 h.

## Supplementary information

Figure S1, Figure S2, Figure S3, Figure S4, Table S1, Table S2, Table S3, Table S4

Table S5

Table S6

Table S7
