# Author Correction: Genome-wide identification of and functional insights into the late embryogenesis abundant (*LEA*) gene family in bread wheat (*Triticum aestivum*)

**DOI:** 10.1038/s41598-020-70133-8

**Published:** 2020-08-04

**Authors:** Hao Liu, Mingyan Xing, Wenbo Yang, Xiaoqian Mu, Xin Wang, Feng Lu, Yao Wang, Linsheng Zhang

**Affiliations:** 1 College of Life Science/State Key Laboratory of Crop Stress Biology for Arid Areas, Northwest A&F University, Yangling, China; 2 Cereal Crops Research Institute, Henan Academy of Agricultural Sciences, Zhengzhou, China; 3 College of Life Science, Jinan University, Guangzhou, China

Correction to: *Scientific Reports*10.1038/s41598-019-49759-w, published online 16 September 2019

This Article contains errors.

In the Results section, the subheading,

“Enhancement of the tolerance of recombinant *E. coli* and yeast cells to salt and heat-”.

should read:

“Enhancement of the tolerance of recombinant *E. coli* and yeast cells to salt and heat-stress”.

Furthermore, in the Results section, under the subheading ‘Determining the endogenous wheat ABA content and expression patterns of *TaLEA* genes under ABA and abiotic stress treatments’,

“To confirm the results of the microarray data, we selected 29 *TaLEA* genes belonging to LEA_1, LEA_2, LEA_3, LEA_4, LEA_5, LEA_6, SMP and three DHN subfamilies to investigate the expression patterns of *TaLEA* genes in wheat seedlings subjected to ABA and abiotic stress treatments by real-time PCR (Fig. 5).”

should read:

“To confirm the results of the microarray data, we selected 22 *TaLEA* genes belonging to LEA_1, LEA_2, LEA_4, LEA_5, SMP and three DHN subfamilies to investigate the expression patterns of *TaLEA* genes in wheat seedlings subjected to ABA and abiotic stress treatments by real-time PCR (Fig. 5).”

In the Methods section, under the subheading ‘In vivo assay of the stress tolerance of transformed *E. coli*’,

“Heat and salt tolerance assays were performed as described previously^48^.”

should read:

“Heat and salt tolerance assays were performed as described previously^26^.”

Finally, this Article contains errors in Figure 5 and Supplementary Table S2, where the primers of TaLEA_3-5, TaLEA_6-1, TaLEA_6-2, TaLEA_6-3, TaSMP8 and TaSMP9 are incorrectly included. In addition, Figure 5 incorrectly includes the primer of TaLEA_3-3, and Supplementary Table S2 incorrectly includes the primer of TaLEA_3-4. The correct Figure 5 and Supplementary Table S2 appear below as Figure 1 and Table 1 respectively.

**Figure 1:**
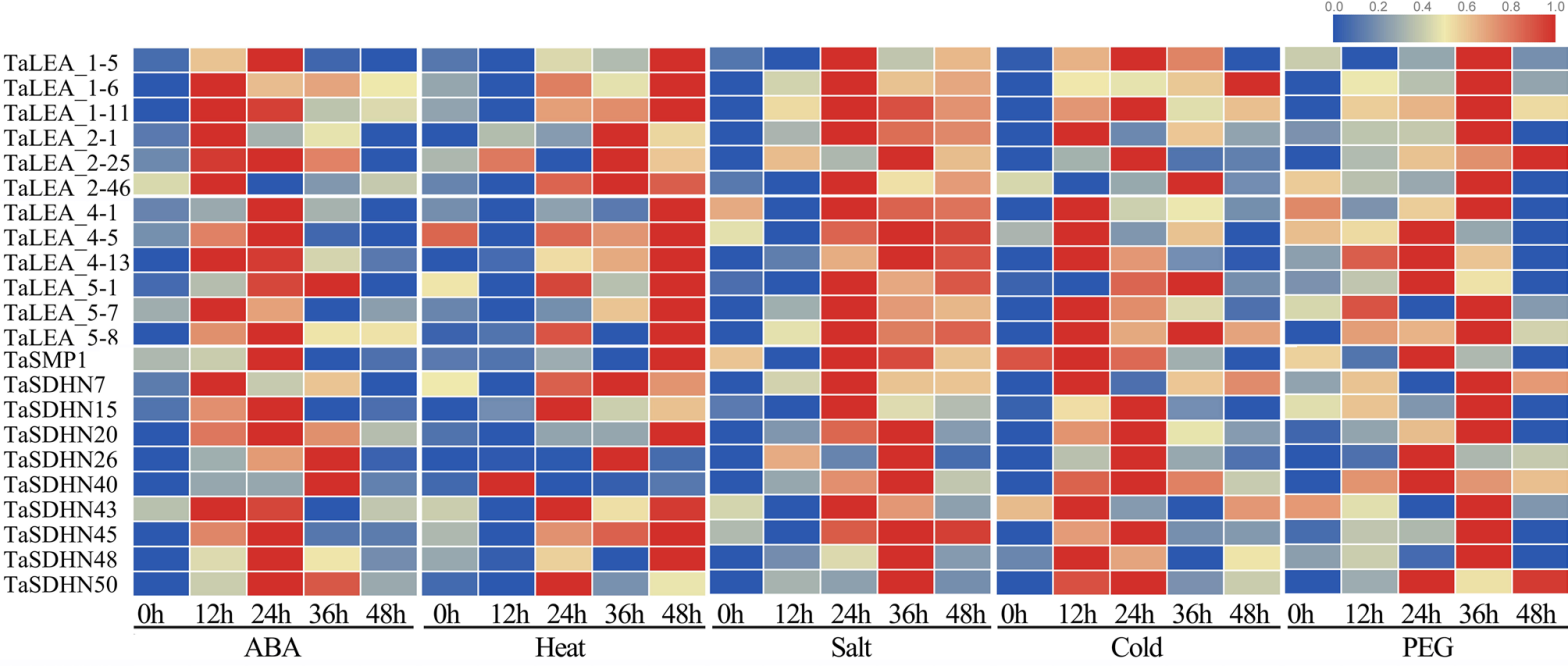
Expression of *TaLEA* genes in response to ABA, heat, NaCl, cold, and PEG treatments determined by real-time PCR. The expression level of wheat *actin* was used as the internal control to standardize the RNA samples for each reaction. The values are the mean ± SE from three samples.

**Table 1:** Real time PCR primers of *TaLEA* genes.

Genes	Primer name	Sequence (5′-3′)
*Actin*	Qactin F	ACCCAACCAGAAACAGCAAC
	Qactin R	CTCATCCAACGAAACGGAAT
*TaLEA_1-5*	QTaLEA_1-5 F	AAGATGAAGGACGGCGCG
	QTaLEA_1-5 R	CTTGTGGATGTGGTGCGAC
*TaLEA_1-6*	QTaLEA_1-6 F	TGTGCATCGATTCCTTCACG
	QTaLEA_1-6 R	GCATGCATGACTCATCGCTT
*TaLEA_1-11*	QTaLEA_1-11 F	GAAGGCCAAGACCAAGATCG
	QTaLEA_1-11 R	CTTGTGGATGCGGTGCTC
*TaLEA_2-1*	QTaLEA_2-1 F	CTCCTTCAACCTCAAGTGCG
	QTaLEA_2-1 R	GATGAGGAAGTCGTAGGGCA
*TaLEA_2-25*	QTaLEA_2-25 F	ACGGAGAGGATGATGAGCAG
	QTaLEA_2-25 R	CGTCGCTCTTGAACGTGTAG
*TaLEA_2-46*	QTaLEA_2-46 F	GTGAGCTTCAAGGGCATGAC
	QTaLEA_2-46 R	GATGAGGAAGTCGTAGGGCA
*TaLEA_4-1*	QTaLEA_4-1 F	AGTACACCAAGGACTCCGC
	QTaLEA_4-1 R	TTTGTGATGGTCTCGGTGGT
*TaLEA_4-5*	QTaLEA_4-5 F	ACACGCAGGACAGGACATAC
	QTaLEA_4-5 R	GTGATCCTCTCGGTGGTGTC
*TaLEA_4-13*	QTaLEA_4-13 F	GAGAAGGCTGGACAGGTGAT
	QTaLEA_4-13 R	GGTCTCGTTGGTCTTGTCCT
*TaLEA_5-1*	QTaLEA_5-1 F	GGAGGAGGGGTACAGTGAGA
	QTaLEA_5-1 R	CTCGTCGATGTCTATGCCCT
*TaLEA_5-7*	QTaLEA_5-7 F	AGGAGAAACTCGCTGAAGGG
	QTaLEA_5-7 R	TGGTCTTGAACTTGGACTCGT
*TaLEA_5-8*	QTaLEA_5-8 F	CTACGAGGCGCAGGAGAAA
	QTaLEA_5-8 R	GACTCATCGTTGGTGCTCAG
*TaSMP1*	QTaSMP1 F	GTCAATATCCCTGGTGGCCT
	QTaSMP1 R	CTTGTCGTCCTTGTTTCGGG
*TaDHN7*	QTaDHN7 F	GTAGCCGAGCAAAAGACTCG
	QTaDHN7 R	CCGTGGTCTGCTCCTTGTTA
*TaDHN15*	QTaDHN15 F	GGCAAAGATGGAGTTCCAAG
	QTaDHN15 R	CAGTATCCCACCGGTCTTGT
*TaDHN20*	QTaDHN20 F	GGAGTACCAGGGACATCAGC
	QTaDHN20 R	CATCCTCAGACGAGCTGGA
*TaDHN26*	QTaDHN26 F	GCGGCAGCTACTTGAGAGTT
	QTaDHN26 R	AACGTCCCGGGTACATACAA
*TaDHN40*	QTaDHN40 F	AGAAGGGCATCATGGAGAAC
	QTaDHN40 R	GTCATTCCAGTGTGTCCCTG
*TaDHN43*	QTaDHN43 F	AGTTACCGGCGAGAACATCA
	QTaDHN43 R	GACTTCCCGTAGTTGCCATC
*TaDHN45*	QTaDHN45 F	CAGCATGGACACACTGGAAT
	QTaDHN45 R	GCAGCTTCTCCTTCACCTTG
*TaDHN48*	QTaDHN48F	CAGTCACAAAGCCAAAGCAA
	QTaDHN48 R	GACCAGCTCCTCCTCCTTCT
*TaDHN50*	QTaDHN50F	GAGCCCGAGGTTAAGAAGGA
	QTaDHN50 R	TGATCACCTCACCGTTGTCA

