# Supplementary material for: Genome-wide identification of and functional insights into the late embryogenesis abundant (LEA) gene family in bread wheat (Triticum aestivum)
Source: Sci Rep. 2019 Sep 16;9:13375. doi: 10.1038/s41598-019-49759-w (PMC6746774; doi:10.1038/s41598-019-49759-w)
Supplement: Supplementary file 1 — Figure S1, Figure S2, Figure S3, Figure S4, Table S1, Table S2, Table S3, Table S4 [file 41598_2019_49759_MOESM1_ESM.docx]

**Manuscript title: Genome-wide identification and functional insights into the** **l****ate embryogenesis abundant (*LEA*) gene** **family in bread wheat (*****Triticum aestivum*)**

**Liu Hao^1^, Xing Mingyan^1^, Yang Wenbo^2^, Mu Xiaoqian^1^, Wang Xin^1^, Lu Feng^3^, Wang Yao^1*^, Zhang Linsheng^1*^**

**1.** **College of life science/State Key Laboratory of Crop Stress Biology for Arid Areas, Northwest A&F University, Yangling, China.**

**2. Cereal Crops Research Institute, Henan Academy of Agricultural Sciences, Zhengzhou, China.**

**3. College of life science, Jinan university, Guangzhou, China.**

***Corresponding author E-mail:** **[wangyao@nwsuaf.edu.cn](mailto:wangyao@nwsuaf.edu.cn);** [**linszhang@nwsuaf.edu.cn**](mailto:linszhang@nwsuaf.edu.cn)**.**

**Supporting information：**

**Figure S1. SDS-PAGE analysis of eight selected LEA genes from *T.aestivum*.** Lane: 1: Marker; 2: +IPTG pET28a; 3: +IPTG TaLEA_1-5; 4: +IPTG TaLEA_2-1; 5: +IPTG TaLEA_3-3; 6: +IPTG TaLEA_4-1; 7: +IPTG TaLEA_5-1; 8: +IPTG TaLEA_6-2; 9: +IPTG TaSMP8; 10: +IPTG TaDHN43.

**Figure S2. Conserved motif and sequence analyses of TaLEA proteins.** All motifs were identified by MEME database with the complete amino acid sequences of TaLEA proteins. The classification of TaLEA proteins were shown different colors.

**Figure S3. Conserved motif analyses in the different groups of TaLEA proteins.** All motifs were identified by MEME database with the complete amino acid sequences of TaLEA proteins. The classification of TaLEA proteins were shown different colors based on the phylogenetic relationship.

**Supplemental Table 1. Clone primers of *TaLEA* genes.**

**Supplemental Table 2. Real time PCR primers of *TaLEA* genes.**

**Supplemental Table 3. Primers used to expression the *TaLEA* genes in *E.coli*.**

**Supplemental Table 4. Primers used to expression the *TaLEA* genes in yeast.**

**Supplemental Table 5. The characteristics of TaLEA proteins.**

**Supplemental Table 6. The *cis*-acting elements related to biotic and abiotic stress responsiveness in the promoter region of *TaLEA* genes**

**Supplemental Table 7. Microarray-based expression pattern of TaLEAs.**


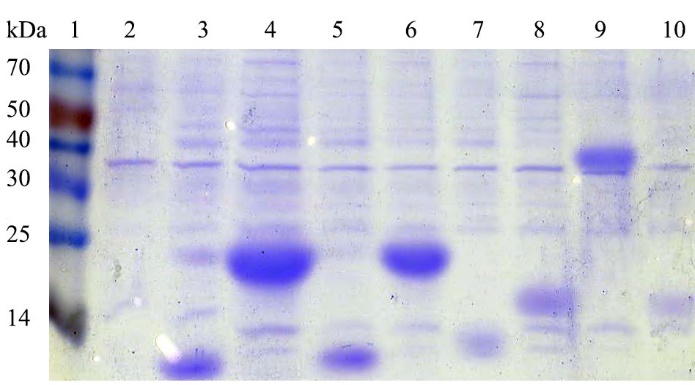


**Figure S1. SDS-PAGE analysis of 8 selected LEA genes from *T.aestivum*.** Lane: 1: Marker; 2: +IPTG pET28a; 3: +IPTG TaLEA_1-5; 4: +IPTG TaLEA_2-1; 5: +IPTG TaLEA_3-3; 6: +IPTG TaLEA_4-1; 7: +IPTG TaLEA_5-1; 8: +IPTG TaLEA_6-2; 9: +IPTG TaSMP8; 10: +IPTG TaDHN43.


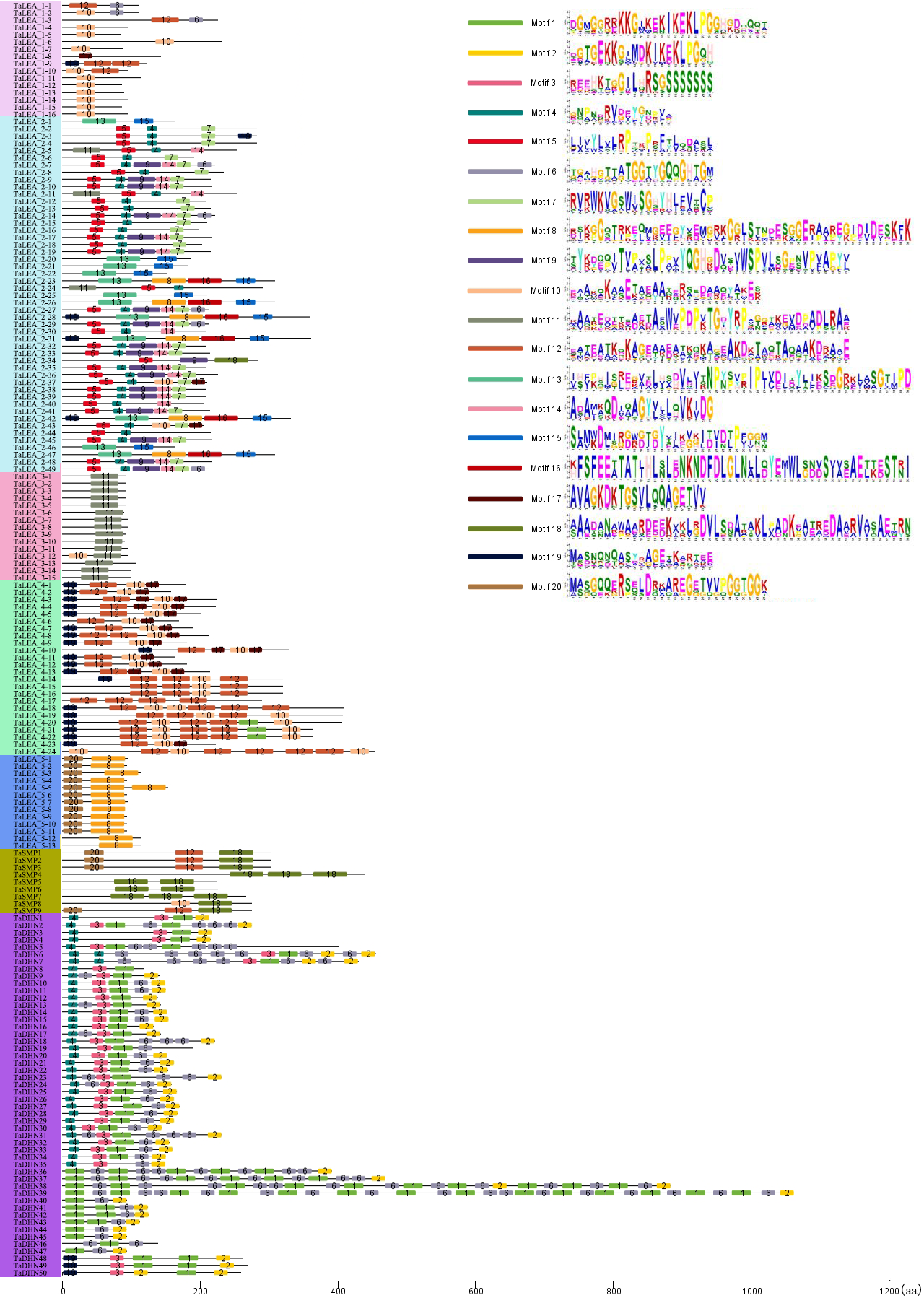


**Figure S2. Conserved motif and sequence analyses of TaLEA proteins.** All motifs were identified by MEME database with the complete amino acid sequences of TaLEA proteins. The classification of TaLEA proteins were shown different colors.


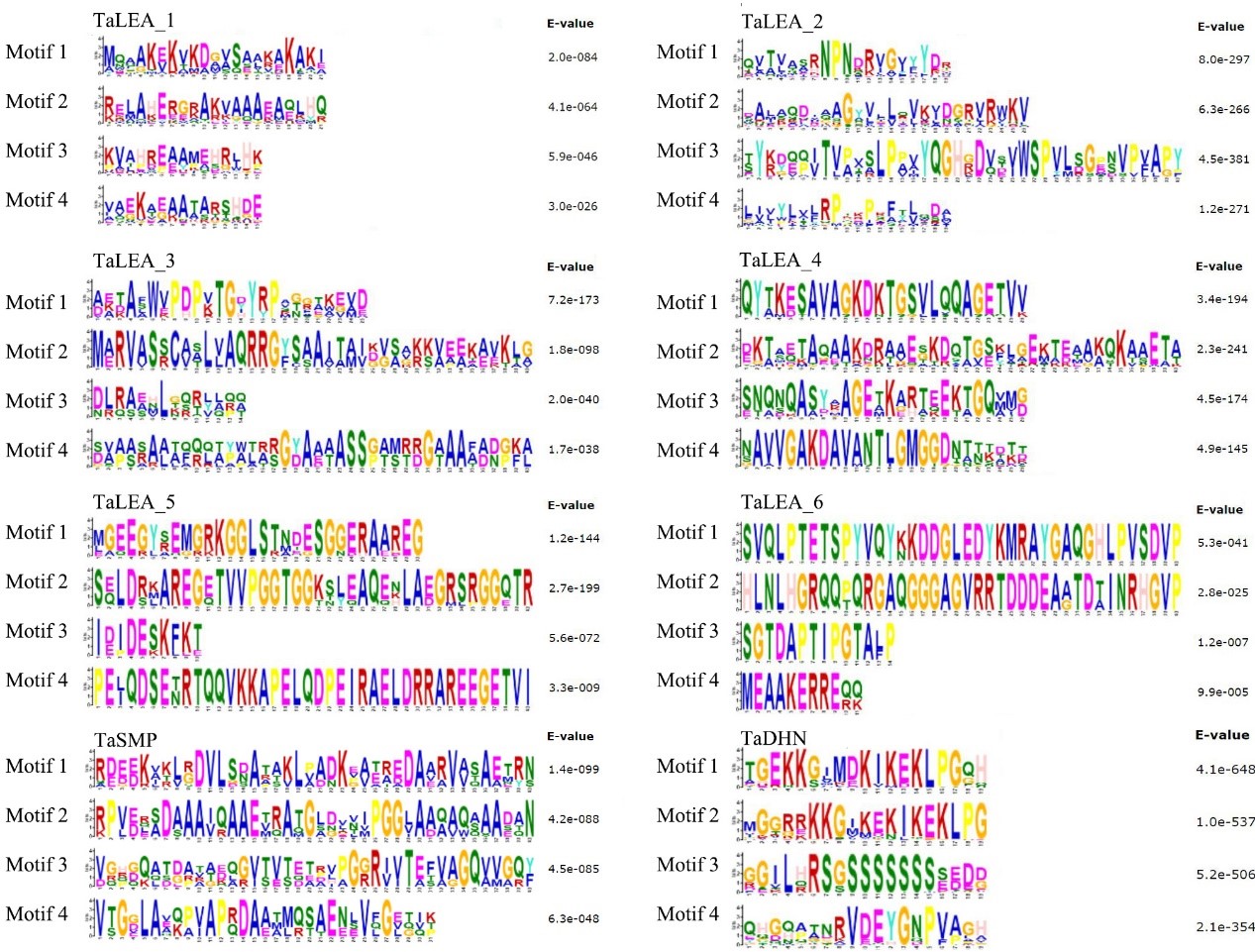


**Figure S3. Conserved motif analyses in the different groups of TaLEA proteins.** All motifs were identified by MEME database with the complete amino acid sequences of TaLEA proteins. The classification of TaLEA proteins were shown different colors based on the phylogenetic relationship.

**Supplemental Table1. Clone primers of *TaLEA* genes.**

| Genes | Primer name | Sequence (5'-3') |
| --- | --- | --- |
| *TaLEA1-1* | TaLEA_1-5 F | ATGGAGCACGCCAAGGAGAAGATGA |
|  | TaLEA_1-5 R | TCAGTGGCCGTGCTTGTGGCCGCCG |
| *TaLEA_2-1* | TaLEA_2-1 F | ATGGCGAGCCTGATGGACAAGGCCA |
|  | TaLEA_2-1 R | CTAGGCGGTGGCCTCCTCGGTCTTG |
| *TaLEA_3-3* | TaLEA_3-3 F | ATCGCCTCCGTTCGTC |
|  | TaLEA_3-3 R | CCGGTAGCGTGTTTGC |
| *TaLEA_4-1* | TaLEA_4-1 F | ATGGCCTCCAACCAGGACAAGGCAA |
|  | TaLEA_4-1 R | CTAGTGGCGGCTCTTTGTGATGGTC |
| *TaLEA_5-1* | TaLEA_5-1 F | GTCGAGCACCTGTCA |
|  | TaLEA_5-1 R | GAGGCACTGAACTGAA |
| *TaLEA_6-2* | TaLEA_6-2 F | ATCGAGCATCCAATCCAGAAA |
|  | TaLEA_6-2 R | CAAGACAGCCTAGCCATGAACTA |
| *TaDHN43* | TaDHN43 F | TCAACGCAAGATGGAGC |
|  | TaDHN43 R | ACCGAGCTTAGTGCTGTCC |
| *TaSMP8* | TaSMP8 F | CATGGCTCAAGCACA |
|  | TaSMP8 R | ACGTGGCAAATTCAA |

**Supplemental Table2. Real time PCR primers of *TaLEA* genes.**

| Genes | Primer name | Sequence (5'-3') |
| --- | --- | --- |
| *Actin* | Qactin F | ACCCAACCAGAAACAGCAAC |
|  | Qactin R | CTCATCCAACGAAACGGAAT |
| *TaLEA_1-5* | QTaLEA_1-5 F | AAGATGAAGGACGGCGCG |
|  | QTaLEA_1-5 R | CTTGTGGATGTGGTGCGAC |
| *TaLEA_1-6* | QTaLEA_1-6 F | TGTGCATCGATTCCTTCACG |
|  | QTaLEA_1-6 R | GCATGCATGACTCATCGCTT |
| *TaLEA_1-11* | QTaLEA_1-11 F | GAAGGCCAAGACCAAGATCG |
|  | QTaLEA_1-11 R | CTTGTGGATGCGGTGCTC |
| *TaLEA_2-1* | QTaLEA_2-1 F | CTCCTTCAACCTCAAGTGCG |
|  | QTaLEA_2-1 R | GATGAGGAAGTCGTAGGGCA |
| *TaLEA_2-25* | QTaLEA_2-25 F | ACGGAGAGGATGATGAGCAG |
|  | QTaLEA_2-25 R | CGTCGCTCTTGAACGTGTAG |
| *TaLEA_2-46* | QTaLEA_2-46 F | GTGAGCTTCAAGGGCATGAC |
|  | QTaLEA_2-46 R | GATGAGGAAGTCGTAGGGCA |
| *TaLEA_3-4* | QTaLEA_3-4 F | ATTTTCCGCCGCGATAACC |
|  | QTaLEA_3-4 R | TCTTGTCCTCCGTCGTGTC |
| *TaLEA_3-5* | QTaLEA_3-5 F | ATTTTCCGCCGCGATAACC |
|  | QTaLEA_3-5 R | TCTTGTCCTCCGTCGTGTC |
| *TaLEA_4-1* | QTaLEA_4-1 F | AGTACACCAAGGACTCCGC |
|  | QTaLEA_4-1 R | TTTGTGATGGTCTCGGTGGT |
| *TaLEA_4-5* | QTaLEA_4-5 F | ACACGCAGGACAGGACATAC |
|  | QTaLEA_4-5 R | GTGATCCTCTCGGTGGTGTC |
| *TaLEA_4-13* | QTaLEA_4-13 F | GAGAAGGCTGGACAGGTGAT |
|  | QTaLEA_4-13 R | GGTCTCGTTGGTCTTGTCCT |
| *TaLEA_5-1* | QTaLEA_5-1 F | GGAGGAGGGGTACAGTGAGA |
|  | QTaLEA_5-1 R | CTCGTCGATGTCTATGCCCT |
| *TaLEA_5-7* | QTaLEA_5-7 F | AGGAGAAACTCGCTGAAGGG |
|  | QTaLEA_5-7 R | TGGTCTTGAACTTGGACTCGT |
| *TaLEA_5-8* | QTaLEA_5-8 F | CTACGAGGCGCAGGAGAAA |
|  | QTaLEA_5-8 R | GACTCATCGTTGGTGCTCAG |
| *TaLEA_6-1* | QTaLEA_6-1 F | TGGAGGACTACAAGATGCGG |
|  | QTaLEA_6-1 R | AGGTTCAGGTGCTCAGTCG |
| *TaLEA_6-2* | QTaLEA_6-2 F | TGGAGGACTACAAGATGCGG |
|  | QTaLEA_6-2 R | AGGTTCAGGTGCTCAGTCG |
| *TaLEA_6-3* | QTaLEA_6-3 F | TGGAGGACTACAAGATGCGG |
|  | QTaLEA_6-3 R | AGATTCAGGTGCTCGGTCG |
| *TaSMP1* | QTaSMP1 F | GTCAATATCCCTGGTGGCCT |
|  | QTaSMP1 R | CTTGTCGTCCTTGTTTCGGG |
| *TaSMP8* | QTaSMP8 F | CGAGCAAGGTGACGATCGG |
|  | QTaSMP8 R | GACCGTGATCTTGTCCTCGT |
| *TaSMP9* | QTaSMP9 F | GGAGGAGGACACGAGCAAG |
|  | QTaSMP9 R | GACCGTGATCTTGTCCTCGT |
| *TaDHN7* | QTaDHN7 F | GTAGCCGAGCAAAAGACTCG |
|  | QTaDHN7 R | CCGTGGTCTGCTCCTTGTTA |
| *TaDHN15* | QTaDHN15 F | GGCAAAGATGGAGTTCCAAG |
|  | QTaDHN15 R | CAGTATCCCACCGGTCTTGT |
| *TaDHN20* | QTaDHN20 F | GGAGTACCAGGGACATCAGC |
|  | QTaDHN20 R | CATCCTCAGACGAGCTGGA |
| *TaDHN26* | QTaDHN26 F | GCGGCAGCTACTTGAGAGTT |
|  | QTaDHN26 R | AACGTCCCGGGTACATACAA |
| *TaDHN40* | QTaDHN40 F | AGAAGGGCATCATGGAGAAC |
|  | QTaDHN40 R | GTCATTCCAGTGTGTCCCTG |
| *TaDHN43* | QTaDHN43 F | AGTTACCGGCGAGAACATCA |
|  | QTaDHN43 R | GACTTCCCGTAGTTGCCATC |
| *TaDHN45* | QTaDHN45 F | CAGCATGGACACACTGGAAT |
|  | QTaDHN45 R | GCAGCTTCTCCTTCACCTTG |
| *TaDHN48* | QTaDHN48 F | CAGTCACAAAGCCAAAGCAA |
|  | QTaDHN48 R | GACCAGCTCCTCCTCCTTCT |
| *TaDHN50* | QTaDHN50 F | GAGCCCGAGGTTAAGAAGGA |
|  | QTaDHN50 R | TGATCACCTCACCGTTGTCA |

**Supplemental Table3. PCR primers used to expression the *TaLEA* genes in *E.coli*.**

| Genes | Primer name | Sequence (5'-3') |
| --- | --- | --- |
| *TaLEA1-1* | EX-TaLEA_1-5 F | ATGGGTCGCGGATCC*GAATTC*ATGGAGCACGCCAAG |
|  | EX-TaLEA_1-5 R | CTCGAGTGCGGCCGC*AAGCTT*TCAGTGGCCGTGCTT |
| *TaLEA_2-1* | EX-TaLEA_2-1 F | ATGGGTCGCGGATCC*GAATTC*ATGGCGAGCCTGATG |
|  | EX-TaLEA_2-1 R | CTCGAGTGCGGCCGC*AAGCTT*CTAGGCGGTGGCCTC |
| *TaLEA_3-3* | EX-TaLEA_3-3 F | ATGGGTCGCGGATCC*GAATTC*ATGGCACGGGTTGTG |
|  | EX-TaLEA_3-3 R | CTCGAGTGCGGCCGC*AAGCTT*TCATTGCTGCTGCAG |
| *TaLEA_4-1* | EX-TaLEA_4-1 F | ATGGGTCGCGGATCC*GAATTC*ATGGCCTCCAACCAG |
|  | EX-TaLEA_4-1 R | CTCGAGTGCGGCCGC*AAGCTT*CTAGTGGCGGCTCTT |
| *TaLEA_5-1* | EX-TaLEA_5-1 F | ATGGGTCGCGGATCC*GAATTC*ATGGCGTCCGGTCAG |
|  | EX-TaLEA_5-1 R | CTCGAGTGCGGCCGC*AAGCTT*CTAGGACTTGGTCTT |
| *TaLEA_6-2* | EX-TaLEA_6-2 F | ATGGGTCGCGGATCC*GAATTC*ATGGAGGCGGCGAAG |
|  | EX-TaLEA_6-2 R | CTCGAGTGCGGCCGC*AAGCTT*CTACGGCACGCCGTG |
| *TaDHN43* | EX-TaDHN43 F | ATGGGTCGCGGATCC*GAATTC*ATGGAGCACCAGGGG |
|  | EX-TaDHN43 R | CTCGAGTGCGGCCGC*AAGCTT*TTAGTGCTGTCCAGG |
| *TaSMP8* | EX-TaSMP8 F | ATGGGTCGCGGATCC*GAATTC*ATGGCTCAAGCACAG |
|  | EX-TaSMP8 R | CTCGAGTGCGGCCGC*AAGCTT*TCAAGCGTCGTCGTC |

**Supplemental Table4. Primers used to expression the *TaLEA* genes in yeast.**

| Genes | Primer name | Sequence (5'-3') |
| --- | --- | --- |
| *TaLEA1-1* | Yeast-TaLEA_1-5 F | AACTAATTATTCGAA*GGATCC*ATGGAGCACGCCAAG |
|  | Yeast -TaLEA_1-5 R | AGGGAATTCTACGTA*GGATCC*TCAGTGGCCGTGCTT |
| *TaLEA_2-1* | Yeast-TaLEA_2-1 F | AACTAATTATTCGAA*GGATCC*ATGGCGAGCCTGATG |
|  | Yeast-TaLEA_2-1 R | AGGGAATTCTACGTA*GGATCC*CTAGGCGGTGGCCTC |
| *TaLEA_3-3* | Yeast-TaLEA_3-3 F | AACTAATTATTCGAA*GGATCC*ATGGCACGGGTTGTG |
|  | Yeast-TaLEA_3-3 R | AGGGAATTCTACGTA*GGATCC*TCATTGCTGCTGCAG |
| *TaLEA_4-1* | Yeast-TaLEA_4-1 F | AACTAATTATTCGAA*GGATCC*ATGGCCTCCAACCAG |
|  | Yeast-TaLEA_4-1 R | AGGGAATTCTACGTA*GGATCC*CTAGTGGCGGCTCTT |
| *TaLEA_5-1* | Yeast-TaLEA_5-1 F | AACTAATTATTCGAA*GGATCC*ATGGCGTCCGGTCAG |
|  | Yeast-TaLEA_5-1 R | AGGGAATTCTACGTA*GGATCC*CTAGGACTTGGTCTT |
| *TaLEA_6-2* | Yeast-TaLEA_6-2 F | AACTAATTATTCGAA*GGATCC*ATGGAGGCGGCGAAG |
|  | Yeast-TaLEA_6-2 R | AGGGAATTCTACGTA*GGATCC*CTACGGCACGCCGTG |
| *TaDHN43* | Yeast-TaDHN43 F | AACTAATTATTCGAA*GGATCC*ATGGAGCACCAGGGG |
|  | Yeast-TaDHN43 R | AGGGAATTCTACGTA*GGATCC*TTAGTGCTGTCCAGG |
| *TaSMP8* | Yeast-TaSMP8 F | AACTAATTATTCGAA*GGATCC*ATGGCTCAAGCACAG |
|  | Yeast-TaSMP8 R | AGGGAATTCTACGTA*GGATCC*TCAAGCGTCGTCGTC |
